# Exosomes: New Insights into the Pathogenesis of Metabolic Syndrome

**DOI:** 10.3390/biology12121480

**Published:** 2023-12-01

**Authors:** Ning Wang, Jing Li, Zixuan Hu, Ebenezeri Erasto Ngowi, Baolong Yan, Aijun Qiao

**Affiliations:** 1 Zhongshan Institute for Drug Discovery, Shanghai Institute of Materia Medica, Chinese Academy of Sciences, Zhongshan 528400, China; wangning@zidd.ac.cn (N.W.); lijing@zidd.ac.cn (J.L.); huzixuan@zidd.ac.cn (Z.H.); ebenezerngowi92@gmail.com (E.E.N.); 2 Shanghai Institute of Materia Medica, Chinese Academy of Sciences, 555 Zu Chong Zhi Road, Shanghai 201203, China; 3 University of Chinese Academy of Sciences, Beijing 101408, China; 4 Department of Biological Sciences, Dar es Salaam University College of Education, Dar es Salaam 2329, Tanzania; 5 School of Basic Medical Sciences, Wenzhou Medical University, Wenzhou 325035, China; 1982ybllog@163.com

**Keywords:** exosome, biogeneration, function, metabolic syndrome

## Abstract

**Simple Summary:**

Patients with long-lasting metabolic syndrome (MS) are at high risk for cancer and complications of cardiovascular diseases, which remain the leading cause of death globally. Despite the development of many novel therapies for MS, a huge unmet need persists. Currently, there is considerable evidence that exosomes are strongly associated with the occurrence and progression of MS. Thus, this article reviews the composition, biogenesis, biological functions and potential applications of exosomes, particularly focusing on current advances about the potential role of exosomes in the development of MS. Comprehensively understanding the effects of exosome on MS could have the significant potential to identify safe and effective diagnostic and therapeutic approaches to MS.

**Abstract:**

Exosomes are a subtype of extracellular vesicles (EVs) with a diameter of 30~150 nm (averaging ~100 nm) that are primarily produced through the endosomal pathway, and carry various components such as lipids, proteins, RNA, and other small molecular substances. Exosomes can mediate intercellular communication through the bioactive substances they carry, thus participating in different physiological activities. Metabolic syndrome (MS) is a disease caused by disturbances in the body’s metabolism, mainly including insulin resistance (IR), diabetes, obesity, non-alcoholic fatty liver disease (NAFLD), hyperlipidemia, and atherosclerosis (AS). Recent studies have shown that exosomes are closely related to the occurrence and development of MS. Exosomes can act as messengers to mediate signaling transductions between metabolic cells in the organism and play a bidirectional regulatory role in the MS process. This paper mainly reviews the components, biogenesis, biological functions and potential applications of exosomes, and exosomes involved in the pathogenesis of MS as well as their clinical significance in MS diagnosis.

## 1. Introduction

EVs are a collective term for vesicles released by cells that have a membrane structure. According to their diameters, they can be simply classified into four types: exosomes (30~150 nm), microvesicles (100~1000 nm), apoptotic bodies (100~5000 nm), and oncosomes (1~10 μm). Exosomes are spherical bilayer vesicles formed by mammalian cells through a series of regulatory processes [1]. They are usually observed in cell culture supernatants, plasma, saliva, urine, amniotic fluid, malignant ascites, and other biological fluids, and are rich in biologically active substances such as proteins, lipids, nucleic acids and metabolites [2,3]. As mediators of cell-to-cell communication via bio-active substances they carry, exosomes exert an important role in the progress of various diseases, such as MS, cardiovascular diseases and neuron diseases [4]. In recent years, exosomes have been extensively studied due to their outstanding biochemical properties, and have emerged as an important means for disease diagnosis and drug delivery because of their biocompatibility, stability, and safety [5,6,7,8]. MS is a series of metabolic diseases caused by the impaired metabolism of various substances, such as proteins, fats, and carbohydrates in the human body. Due to lack of effective diagnostic methods and therapeutic strategies, the disease has progressed to become one of the most threatening public health problems affecting human health and quality of life. Recent documents have shown that the biological activity of exosomes is tightly related to the development of MS, and thus may have potential application for clinical diagnosis and treatment of MS. This review summarizes the biogenesis, biological characteristics, and potential application of exosomes, as well as the role of exosomes in MS, with the aim of providing some data references on the latest developments in research involving exosomes.

## 2. Biogenesis of Exosomes

Exosome biogenesis involves three steps: intraluminal vesicles (ILVs) production, multivesicular bodies (MVBs) transportation, and exosomes release [9].

### 2.1. ILVs Production Involves Two Mechanisms according to Their Reliance on Endosomal Sorting Complex Required for Transport (ESCRT), Hence Referred to as ESCRT-Dependent and -Independent Pathways

**Figure 1:**
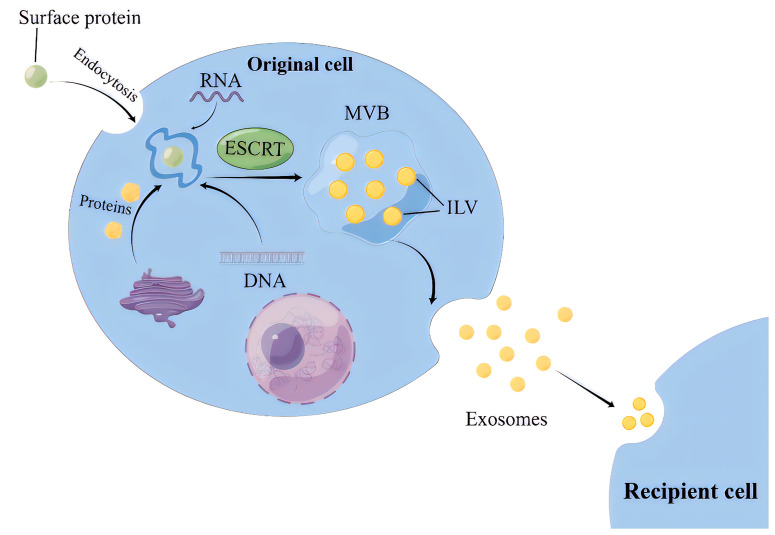
The process of exosomes intracellular biogenesis and secretion. MVB, Multivesicular body; ESCRT, Endosomal sorting complexes required for transport; ILV, intraluminal vesicles.

For the ESCRT-dependent pathway, the initial stage involves the capture of ubiquitinated proteins by the ESCRT-0 complex on the cell membrane, thereby concentrating cargoes (such as DNA, RNA, and proteins) on the membrane [10]. While the cargoes are aggregated, ESCRT-I and ESCRT-II complexes are recruited to the cell membrane, leading to membrane protrusion and the initial formation of intraluminal vesicles (ILVs) [11]. Subsequently, the ubiquitinated cargoes interact with the ESCRT-I component TSG101 and the HRS protein in ESCRT-0 to be further concentrated and immobilized on the membrane [12]. Finally, the ILVs maturation is promoted by the ESCRT-III complex, VPS4 protein, and auxiliary protein VTA1, which are able to provide energy. Thereafter, ILVs accumulate inside the cells and develop into multivesicular bodies (MVBs) (Figure 1) [13].

The non-ESCRT-dependent pathway involves several mechanisms, one of which is the ceramide-dependent mechanism [14]. Ceramide is formed by the hydrolysis of sphingomyelin phosphatidylcholine, which can induce the aggregation of small microdomains into larger domains, promoting domain-induced budding. It has been reported that the transfer of EVs-related domains into endosomal cavities depends on sphingolipid ceramide in mouse glial cells [15]. Another mechanism is the tetraspanins-dependent pathway, characterized by a family of proteins with four transmembrane domains. In mammals, this family’s members have as many as 33 proteins, including CD9, CD37, CD51, CD53, CD63, CD81, CD82, and others [16]. Study has shown that CD63 is required for ILV production and subsequent exosomes release [17,18]. In addition, evidence has shown that Rab proteins-mediated pathway promotes the fusion of exosomes with the cell membrane and the release of exosomes via the interaction of Rab31 and Rab27, and their effector proteins [19]. 

### 2.2. MVBs Transportation and Exosomes Release

MVBs transportation from the site of generation to the cell membrane occurs mainly through a network of microtubules, where small GTPases (e.g., Rab proteins) play a key role [20]. The fusion of MVBs with the cell membrane is mediated by the soluble N-ethylmaleimide sensitive factor attachment receptor (SNARE) protein complex. The exosome transport and release are influenced by a variety of organelles, mainly lysosomes, which can significantly reduce the release of exosomes once it fuses with MVBs [21]. Apart from lysosomes, the golgi apparatus, while not directly involved in exosome biogenesis, participates in the post-translational modification and trafficking of proteins destined for exosomes [22]. In addition, mitochondrial stress and dysfunction appear to influence exosome production and content, but the precise mechanisms are not clear [23,24,25].

## 3. Biological Characteristics of Exosomes

**Figure 2:**
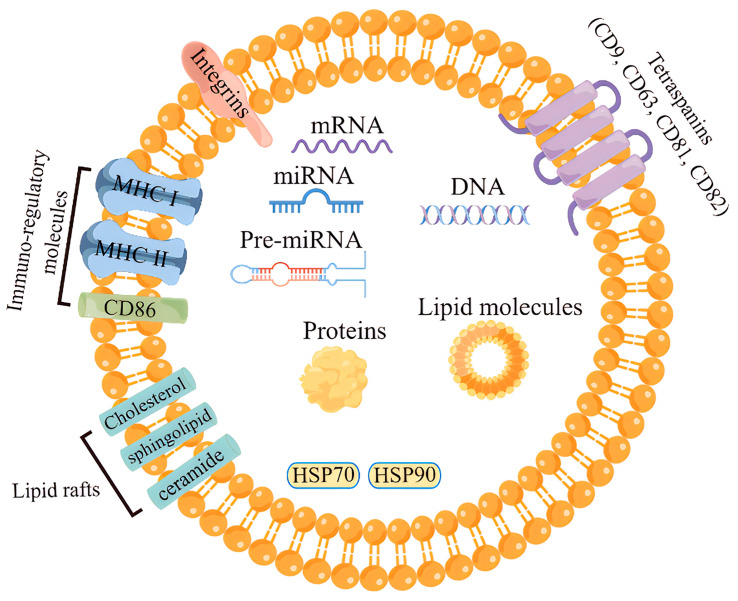
The structure and biological composition of exosome.

The outer membrane of exosomes is rich in cholesterol, sphingolipids, ceramides, glycolipids, and glycerophospholipid chains, which together play crucial roles in the cellular microenvironment. The main roles of the lipid components in the exosome membrane are to provide some stiffness, ensure bioavailability, and prevent the bioactive materials carried from degradation. Moreover, evidence have shown that exosomes lipids are also involved in the transport of lipids in vivo [26,27]. Different cells produce different exosomes that carry similar conserved proteins on their surface alone, such as the major histocompatibility complex MHC I and MHC II molecules, heat-shock proteins, four transmembrane proteins (CD9, CD63, CD81), integrins, cytoskeletal proteins and some biological enzymes [4,28,29,30]. The inclusions of exosomes mainly include proteins, including cytokines, miRNA, mRNA, DNA, and lipids. Current research from 286 exosomes studies have reported approximately 41,860 proteins, 7540 RNAs and 1160 lipid molecules from exosomes released from various cell types (Figure 2) [31,32,33].

The composition of the exosome inclusions of different cellular sources and states is in a state of dynamic change. The highest proportion of exosomes RNAs are miRNAs [34], which is a unique feature that distinguishes exosomes from other extracellular vesicles. The types and amount of RNAs present in the exosome reflect the secretory-cell types and its physiological/pathological state [35]; however, the types and expression levels of these RNAs differ significantly from these secretory cells [36], suggesting that there is a sorting mechanism for RNA assembly in exosomes, although the exact mechanism remains elusive.

## 4. Exosome Separation and Technical Challenges 

Several prominent methods have been established and utilized for exosome extraction and separation, encompassing ultracentrifugation, size-exclusion chromatography, ultrafiltration, immunomagnetic bead separation, and microfluidic chip technology. Ultracentrifugation is the most prevalent technique and is highly regarded for its simplicity and affordability. However, ultracentrifugation tends to require longer processing times, with higher losses and lower purity due to centrifugal forces, thus having an impact on subsequent identification [37,38]. Size-exclusion chromatography is the second most used technique after ultracentrifugation and offers a simple operation for exosome extraction while preserving biological activity, making it suitable for experiments of various scales and effective in isolation of large exosomes. However, it also presents some limitations, such as sample loss, limited separation efficiency, time cost, and unsuitable for the extraction of small exosomes [39]. Extraction of exosomes using ultrafiltration is high yielding, simple and fast. Nevertheless, it is prone to clogging the membrane pores and contaminating large protein particles, resulting in lower exosome recovery as well as purity [40]. The immunomagnetic bead separation, grounded in the principle of antigen–antibody binding, can procure remarkably pure exosomes. However, it comes at an elevated cost and demands advanced technical proficiency [41]. Conversely, microfluidic chip technology facilitates swift and efficient exosome separation even from minimal sample quantities. Still, it is often encumbered by heightened technical, financial, and instrumental prerequisites [42]. While current exosome separation methodologies have achieved a level of maturity, formidable challenges persist. First, exosomes are ubiquitously present in a range of biological fluids, including blood, urine, and saliva, adding layers of complexity to the separation process. Second, the intricacies of existing techniques, compounded by the absence of a universally accepted standardization, could potentially give rise to inconsistencies in research findings across diverse research settings. Third, we have to acknowledge the difficulty of separating exosomes from other types of extracellular vesicles by utilizing single technique, thereby precluding a definite attribution of a particular function of the different types of secreted vesicles [43]. Therefore, these elements, while influencing the efficacy of exosome separation, also potentially impede the fidelity and comparability of exosomes studies. In light of these challenges, the scientific community remains undeterred to innovate more adept, economical, and standardized protocols for exosome extraction and separation, thereby fueling the advancement of exosome studies [44,45,46].

## 5. Biological Functions of Exosomes

Exosomes are natural transport carriers, and their inner lumen can be loaded with various biomolecules, such as proteins and nucleic acids. Exosomes also embed and anchor various protein ligands on their surface, which upon recognition and binding to the recipient cells can lead to reactive changes, thus affecting intracellular signaling pathways and the physiological state of the recipient cells. Therefore, exosomes can serve as carriers for biological molecules and signals for communication between cells. 

Different cells achieve intercellular communication by secreting exosomes carrying different components, and these exosomes are taken up by the recipient cells to exchange molecules or signals through substance exchange and release of endosomes, thus leading to the subsequent influence of recipient cells behavior and phenotype features. The intercellular communications mainly undergo via the following mechanisms. First, exosomes surface proteins can modulate the signaling pathways of target cells via directly binding to its cell receptors; Jodo et al. showed that the membrane of exosomes secreted by T cells contains a signaling molecule, Fas L, which specifically binds to transmembrane protein, Fas, on the receptor cell membrane and induces its trimerization, thus forming an apoptosis-inducing complex to initiate cell death [47]. Second, exosomes can fuse with cell membranes and deliver functional proteins, miRNAs, mRNAs, and other biomolecules to target cells. Montecalvo et al. identified and experimentally demonstrated a way for exosomes contents to enter recipient cells via exosome–cell membrane fusion [48]. In target cells, mRNAs from exosomes can translate into corresponding proteins; meanwhile, miRNAs and siRNAs can directly regulate cellular processes by regulating the expressions of target genes. Third, target cells engulf exosomes via endocytosis, and exosomes can be re-released in target cells or degraded via the lysosomal pathway [49,50]. The intercellular communication function of exosomes allows them to play a key role in a variety of physiological processes. It has been shown that neuronal cells can regulate the expression of glutamate transporter-1 (GLT-1) in astrocytes by secreting exosomes carrying miR-124-3p, thereby affecting the physiological state of astrocytes and maintaining the normal function of the nervous system [51]. Mesenchymal stem cell-derived exosomes are also able to effectively deliver therapeutic molecules into cardiomyocytes to promote heart healing from myocardial infarction [52]. Moreover, cancer cell-derived exosomes are capable of transmitting signals and transporting substances over long distances, thus enabling material exchange and communication between cells which induce cell proliferation and metastasis [53]. Therefore, exosomes show a wide range of potential applications (i.e., biomarkers, drug delivery, gene therapy, and tissue repair) in the biomedical field due to their unique biological properties.

## 6. Application of Exosomes

Due to their excellent properties, exosomes play an extremely important role in disease diagnosis and treatment. First, given their presence in fluids like blood, urine, and saliva, exosomes can be isolated using liquid biopsy, providing a non-invasive strategy alternative to traditional biopsies [54], Second, the compositions of exosomes can be analyzed by mass spectrometry and other analytical methods to obtain a large amount of information about molecular carriers inside and outside the cells, on the basis of which a comprehensive diagnosis can be achieved at different states of disease progress. It was reported that 120 plasma exosome samples collected from the patients were used to screen the specific biomarkers of 16 different types of tumors by proteomic analysis. The results show that they achieved 95% sensitivity and 90% specificity for the classification of various tumors [55]. EPI-CE kit (Exosome dx company) and ExoDx Prostate-IntelliScore Diagnostics Product (Bio-Techne company) have been developed and marketed for the diagnosis of prostate cancer by screening RNA biomarkers originating from prostate-specific or urine exosomes [56,57]. Third, exosomes are natural carriers with the advantages of long half-life and natural non-toxicity. They also have the unique ability to target homing and deliver the substances to the target cells. The biological nature of exosomes allows them to easily cross the blood–brain barrier, making them an ideal cargo for drug delivery into the brain. For instance, exosomes can bind to an anti-CD22 monoclonal antibody fragment (CD22-F (ab’) 2) and wrap doxorubicin (DOX) to form CD22-F (ab’) 2-Exo-DOX, which can penetrate the BBB and accurately deliver DOX to lymphoma cells of primary central nervous system, thus enhancing anti-tumor activity in tumor-bearing mice [58]. Collectively, the above evidence indicate that exosomes have high potential in diagnosing and treating diseases, including those located in deep regions that are not easily reached. 

## 7. Exosomes Involved in MS Progress

MS is a complex syndrome of metabolic disorders, including IR, diabetes mellitus (DM), obesity, NAFLD, hyperlipidemia, AS and hypertension [59]. The study showed that exosomes play a vital role in the occurrence and development of MS and its related diseases [60]. In this section, we will review the role of exosomes in the progression of metabolic diseases, and the underlying mechanisms by which exosomes regulate systemic energy homeostasis by affecting metabolism in target cells. This would greatly help to understand the etiology of MS better and develop a novel therapeutic strategy for the treatment of MS.

### 7.1. IR

**Figure 3:**
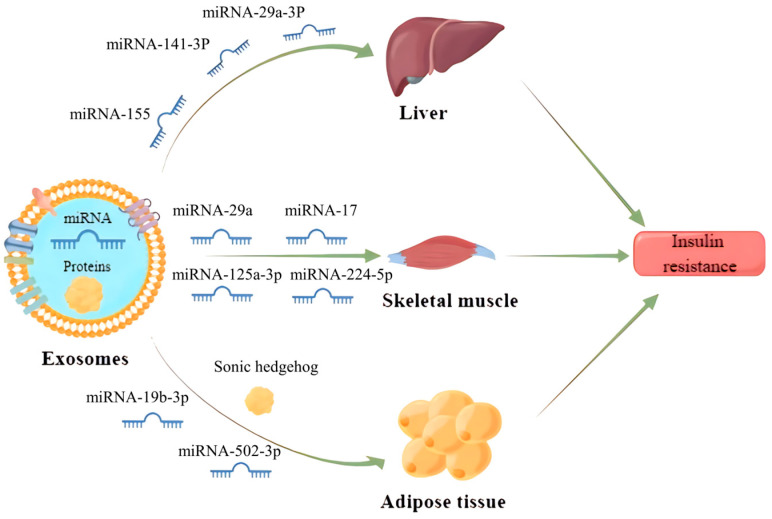
Exosomes released from parent cells promote IR in the liver, skeletal muscle and adipose tissue by delivering the miRNAs and proteins they carry to receipt cells.

IR is a central factor contributing to MS and a driving force for the development of cardiovascular complications associated with type 2 diabetes mellitus (T2DM) and hypertension [61]. It has been found that exosomes can enter the receptor cells by wrapping different inclusions to regulate the transduction of the insulin signaling pathway, which contributes to IR development (Figure 3). Exosomes carrying miR-155 secreted by adipose tissue macrophages into hepatocytes can downregulate glucose transporter-4 (Glut-4) by targeting peroxisome proliferator-activated receptor γ (PPARγ), which ultimately leads to a decrease in insulin sensitivity in the liver and exacerbates the process of diabetes in obese mice [62]. Similarly, miRNA-141-3p, contained in exosomes secreted from adipose tissue and taken up by hepatocytes, could also significantly reduce insulin sensitivity in high-fat fed mice [63]. Apart from that, exosomes can also regulate insulin sensitivity by directly acting on the insulin receptor. For example, adipose-derived exosomes miR-29a-3p in the Zucker diabetic rat significantly aggravates IR in the liver by downregulating the expression of insulin receptor substrate-1 and its phosphorylated levels [64]. 

Gestational diabetes mellitus is also one of the more studied types of diabetes mellitus and its relationship with exosomes has recently received more attention. It has been reported that exosomes miRNA-125a-3p and miRNA-224-5p released from the placenta of patients with gestational diabetes mellitus can effectively regulate the expression of glypican and CD40, and inhibit the PI3K/AKT/Glut-4 signaling axis in skeletal muscle, thus resulting in diabetic IR [65]. In addition, exosomes also play a critical role in the development of IR in adipose tissue. Exosomes miRNA-29b-3p derived from bone marrow mesenchymal stem cells (BM-MSCs) can induce IR in adipose tissue by suppressing sirtuin 1 [66]. Adipocyte-derived exosomes carrying sonic hedgehog can also elicit M1 polarization in macrophages to promote IR in adipose tissue via the Ptch/PI3K pathway [67]. In summary, those studies open a new avenue toward the role of exosomes in the pathogenesis of IR, more complementary studies should pay more attention to unravel the mechanism and the effect of exosomes in the context of IR in humans. 

### 7.2. DM and Its Related Complications

DM is a long-term systemic chronic metabolic disease, mainly classified into type 1 diabetes mellitus (T1DM) and T2DM. Recent studies suggest that miRNAs in exosomes may serve as new diagnostic markers and therapeutic interventions for DM. The levels of circulating exosomes miR-15a, miR-375, miR-126, miR-1, and miR-133 are altered during the early stages of diabetes in rodent and human models and could be potential diagnostic markers for the disease [68]. From a therapeutic point of view, exosomes miR-106b-5p and miR-222-3p released from BM cells can effectively repair damaged pancreatic β-cells in T1DM mice [69]. Exosomes miR-29 secreted by β-cells during the pre-DM phase contributes to the induction of an inflammatory response in macrophages and monocytes, leading to IR and progression of T2DM [70]. Moreover, natural killer cell-derived exosomes miR-1249-3p have been reported to efficiently attenuate IR and inflammation via SKOR1-SMAD6-TLR4-NF-κB axis in adipocytes and hepatocytes, thereby improving glucose metabolism in a T2DM mouse model [71]. An interesting study has suggested that exosomes containing BAY55-9837 injected into diabetic patients can significantly increase insulin secretion and alleviate hyperglycemia, pointing out a promising therapeutic strategy for T2DM treatment [72]. 

Exosomes are also involved in the course of many diabetic complications, including diabetic peripheral neuropathy (DPN), diabetic kidney disease (DKD), diabetic foot ulcers (DFU) and diabetic cardiomyopathy (DCM). It has been shown that Schwann cell-derived exosomes miR-21 can regulate neurite growth in DPN rats by affecting the AKT signaling pathway, opening up new perspectives for the prevention and treatment of DPN [73]. Exosomes derived from renal tubular epithelial cells can promote macrophage glycolysis, renal inflammation, and fibrosis by upregulating HIF-1α expression, which accelerates the progression of DKD [74]. RNAs or proteins in MSCs-derived exosomes (MSCs-Exos) can play a cell-homing role and induce cell proliferation and differentiation, thereby reducing immune responses and promoting cellular self-repair as well as tissue regeneration after injury [46,75]. MSCs-Exos have shown great potential in promoting wound healing and are therefore widely studied for the treatment of DFU [76]. A recent study has demonstrated that exosomes derived from pioglitazone-pretreated MSCs could improve the angiogenic capacity of HUVECs in a high-glucose injury environment by activating the PI3K/AKT/eNOS pathway, thus accelerating wound healing in DFU [77]. Likewise, epidermal stem cell-derived exosomes can enhance diabetic foot ulcer wound healing by reducing inflammation and increasing cell proliferation and angiogenesis [78]. It has also been reported that exosomes miR-126-5p secreted from human umbilical vein endothelial cells (HUVECs) can specifically inhibit the BMP-Smad signaling axis, thereby attenuating vascular calcification in DM mice [79]. MSC-derived exosomes administration can alleviate diabetes-induced myocardial injury and fibrosis in mice by inhibiting the TGF-β1/Smad2 signaling axis [80]. 

Gestational diabetes (GDM) is the most common metabolite disorder during pregnancy and has a complex etiology involving a variety of factors such as genetics and environment. Although most GDM returns to normal after delivery, metabolic abnormalities during pregnancy can still pose serious health risks to the mother, and people with GDM have a higher risk of developing T2DM after delivery than their opposite counterpart [81]. It has been shown that plasma exosomes are significantly higher in pregnant women with GDM than in controls, and the condition might be associated with the enhanced release of inflammatory cytokines from endothelial cells, although the exact mechanism involved is still unclear [82]. Another study focusing on pregnant women with normal glucose tolerance and women with GDM found that treatment of skeletal muscle cells with exosomes isolated from chorionic villous explants of pregnant women with GDM significantly reduces insulin sensitivity and glucose uptake [65].

### 7.3. Obesity

Obesity is one of the most common metabolic diseases, and the prevalence of obesity has greatly increased in past decades in the world [83]. Evidence have demonstrated that circulating exosomes miRNAs primarily secreted by adipose tissue exhibit different profiles between obese patients and healthy individuals [84], and exosomes play a vital role in the exchange of information between adipose tissue and other tissues, thereby contributing to the pathogenesis of obesity and its related diseases [85]. Adipocyte-derived exosomes miR-27a can regulate the hepatic lipid synthesis pathway by inhibiting PPARγ [86]. Exosomes also play an important role in lipid transport by regulating the expression of classical lipid transporters (e.g., ABCA1, ABCG1, LDLR, CD36), where plasma exosomes miR-30e and miR-92a can disrupt lipid metabolism and cause inadequate cholesterol efflux by inhibiting ABCA1 and ABCG1 [87]. However, an opposite observation is that circulating exosomes miRNAs in plasma can suppress lipid degradation in white adipose in obesity-related patients by downregulating the expression of the transcription factor PPARα [88]. Collectively, exosomes play an important role in regulating lipid synthesis as well as lipid transport, and thus could be a good guide for the control of obesity.

### 7.4. NAFLD

NAFLD, like obesity and diabetes, is a highly prevalent global metabolic disease that affects about a quarter of the global adult population and poses a serious health burden [89]. There is growing evidence that exosomes are involved in the development and occurrence of NAFLD [90]. The severity of liver inflammation in NAFLD patients positively correlates with the level of toxic hepatocyte-derived exosomes miR-192-5p [91], indicating that exosomes miR-192-5p can be used as a molecular marker for NAFLD. The release of miR-223-rich exosomes from macrophages can inhibit the expression of transcriptional activator with PDZ-binding motif (TAZ) in hepatocytes, thereby alleviating the progression of NAFLD to NASH and even liver fibrosis [92]. Cheng et al. reported that miR-627-5p from human umbilical cord-derived MSC exosomes could improve NAFLD by reducing the expression of genes related to fat mass and obesity [93]. Interestingly, human liver stem cell-derived exosomes (HLSC-Exos) can significantly attenuate diet-induced steatohepatitis in mice by reducing liver fibrosis and inflammatory responses, and improving histological abnormalities [94]. 

### 7.5. Hyperlipidemia and AS 

Hyperlipidemia is caused by higher percentages of fatty droplets and oxidized low-density lipoprotein (Ox-LDL) in blood composition. Such substances can increase the viscosity of the blood, which leads to a decrease in the oxygen-carrying capacity of the blood and promotes damage to the mucous membranes on the walls of blood vessels, resulting in the formation of atheromatous plaques and AS. It has been shown that macrophage-derived exosomes miR-223 promotes macrophage differentiation, inflammatory response, and disturbs lipid metabolism in adipose tissue, thus exacerbating the progression of AS [95,96]. Interestingly, mice treated with exosomes secreted by Ox-LDL-treated human umbilical vein endothelial cells lead to hyperlipidemia, local inflammation, and the formation and worsening of AS plaques by upregulating the expression of metastasis-associated lung adenocarcinoma transcript 1 (MALAT1) [97]. Visceral adipose tissue-derived exosomes delivery in ApoE-/- mice can augment the hyperlipidemic and promote rapid progression of AS by inducing M1 macrophage polarization via modulation of NF-κB activity [98]. miRNA-99a/146b/378a-rich exosomes secreted from bone marrow-derived macrophages have been demonstrated to suppress inflammation by targeting NF-κB and TNF-α signaling pathways and promoting M2 polarization in recipient macrophages, thereby reducing intraplaque inflammation levels and delaying the AS progression in ApoE-/- mice. Moreover, those exosomes can also reduce excessive hematopoiesis in the bone marrow by reducing the number of circulating myeloid cells and macrophages in aortic root lesions [99]. However, another study demonstrated that miRNAs in platelet exosomes can induce platelet aggregation and mediate the coagulation pathway, thereby promoting the formation of AS [100]. Similarly, it was also reported that miRNAs in exosomes can facilitate the progression of vascular-related diseases [101]. Vascular endothelial cell-derived exosomes miR-126, miR-214 and miR-155 have been identified to play a significant role in promoting neovascularization and maintenance of vascular endothelial cell’s function [102]. Together, miR-126, miR-214, and miR-155 may be exploited as novel therapeutic targets for the prevention and treatment of vascular diseases. Additionally, circulating exosomes miRNA-150 was identified to be associated with the development of vascular inflammation and AS, and may be used as a marker to closely determine the development of related diseases [103]. Meanwhile, a recent study indicated that plasma samples from AS patients showed reduced levels of exosome-derived vitamin D binding protein [104]. 

### 7.6. Hypertension

Hypertension is a complex multifactorial disease, primarily attributable to the interaction between genetic and environmental factors [105]. There is emerging evidence showing that exosomes play a key role in the progression of hypertension. A study has revealed that miRNAs and proteins contained in exosomes derived from endothelial cells and immune cells drastically elicit VSMC proliferation and migration, leading to vascular remodeling, a core mechanism in the etiology of hypertension [106]. Specific miRNAs, such as miR-155, miR-21, miR-34a, miR-145, and miR-22, present in circulating exosomes have been demonstrated to play a crucial role in the pathogenesis of hypertension. They can modulate vascular dilation and constriction by carrying or altering the expression of endothelial nitric oxide synthase (eNOS), thereby participating in blood pressure regulation [107]. Certain miRNAs, like miR-21 and miR-34a within circulating exosomes, have been suggested to regulate salt–water balance and blood pressure homeostasis by impacting renal tubular sodium reabsorption. Additionally, miR-145 within circulating exosomes is believed to affect the release of renin, the activation of angiotensin II, and the secretion of aldosterone, adding another layer of complexity to the regulation mechanism of hypertension [108].

## 8. Conclusions and Future Perspective

Exosomes have been extensively studied in past decades and are demonstrated to play a critical role in the development and progression of metabolic diseases. In this paper, we mainly review the origin of exosomes, the mechanism of their production in vivo, their biological functions, and their relationship with MS, and aim to enhance our understanding of the roles played by exosomes in cellular communication and disease progression. This knowledge could have significant implications for the development of innovative strategies for the diagnosis, treatment, and management of MS and related conditions. Indeed, while significant progress has been made in understanding the biological functions of exosomes, further research is still necessary to unravel the detailed mechanism underlying the interactions between exosomes and cells, and to refine the classification of exosomes based on their contents. The field of exosome research is continually evolving, and ongoing investigations should be attentive to address these knowledge gaps: (1) specific mechanisms through which exosomes communicate with target cells; (2) cargo sorting and packaging; (3) functional heterogeneity of exosomes derived from different cell types or under distinct physiological or pathological conditions; (4) technique challenges for isolation of specific cell-type derived exosomes; (5) standardization of isolation and characterization methods of exosomes; and (6) developing and taking advantages of new technologies to unravel the complexities of exosome biology, such as high-resolution microscopy, omics technologies (proteomics, genomics, metabolomics), and single-vesicle analysis.

## Data Availability

Not applicable.
